# Medication adherence prediction through temporal modelling in cardiovascular disease management

**DOI:** 10.1186/s12911-022-02052-9

**Published:** 2022-11-29

**Authors:** William Hsu, James R. Warren, Patricia J. Riddle

**Affiliations:** School of Computer Science, University of Auckland, Auckland, New Zealand

**Keywords:** Pharmacoepidemiology, Medication adherence, Cardiovascular disease, Deep learning, Recurrent neural networks, Machine learning

## Abstract

**Background:**

Chronic conditions place a considerable burden on modern healthcare systems. Within New Zealand and worldwide cardiovascular disease (CVD) affects a significant proportion of the population and it is the leading cause of death. Like other chronic diseases, the course of cardiovascular disease is usually prolonged and its management necessarily long-term. Despite being highly effective in reducing CVD risk, non-adherence to long-term medication continues to be a longstanding challenge in healthcare delivery. The study investigates the benefits of integrating patient history and assesses the contribution of explicitly temporal models to medication adherence prediction in the context of lipid-lowering therapy.

**Methods:**

Data from a CVD risk assessment tool is linked to routinely collected national and regional data sets including pharmaceutical dispensing, hospitalisation, lab test results and deaths. The study extracts a sub-cohort from 564,180 patients who had primary CVD risk assessment for analysis. Based on community pharmaceutical dispensing record, proportion of days covered (PDC) $\ge$ 80 is used as the threshold for adherence. Two years (8 quarters) of patient history before their CVD risk assessment is used as the observation window to predict patient adherence in the subsequent 5 years (20 quarters). The predictive performance of temporal deep learning models long short-term memory (LSTM) and simple recurrent neural networks (Simple RNN) are compared against non-temporal models multilayer perceptron (MLP), ridge classifier (RC) and logistic regression (LR). Further, the study investigates the effect of lengthening the observation window on the task of adherence prediction.

**Results:**

Temporal models that use sequential data outperform non-temporal models, with LSTM producing the best predictive performance achieving a ROC AUC of 0.805. A performance gap is observed between models that can discover non-linear interactions between predictor variables and their linear counter parts, with neural network (NN) based models significantly outperforming linear models. Additionally, the predictive advantage of temporal models become more pronounced when the length of the observation window is increased.

**Conclusion:**

The findings of the study provide evidence that using deep temporal models to integrate patient history in adherence prediction is advantageous. In particular, the RNN architecture LSTM significantly outperforms all other model comparators.

## Background

The management of CVD risk is necessarily longterm. Such management typically involves disease modifying life style adjustments such as smoking cessation, weight management, diet and physical activity as well as long-term drug therapy for patients assessed to be above the threshold for pharmacological intervention. Studies in the United States have found that among adult populations only a small fraction (2% and 3%) maintain a healthy lifestyle as indicated by nonsmoking, ideal BMI, daily consumption of fruits and vegetables and regular physical exercise [1, 2]. This signals that from a population health perspective, it is insufficient to rely on lifestyle intervention alone in CVD risk management. For patients assessed to be above the threshold for pharmacological treatment, effective drugs exist [3]. Lipid-lowering treatment forms a key component of CVD management and statins are the preferred lipid-lowering drug [4, 5] with strong evidence of effectively lowering CVD risk in primary and secondary prevention [3, 6]. However, maintaining adherence to long-term medication presents a significant challenge to patients benefiting from treatment, with non-adherence associated with risk of major adverse cardiovascular events and mortality [7, 8].

Medication adherence is defined by the International Society for Pharmacoeconomics and Outcomes Research (ISPOR) as “the extent to which a patient acts in accordance with the prescribed interval and dose of a dosing regimen.” [9]. Commonly used CVD medications such as antiplatelets, statins, beta blockers, ACE inhibitors and angiotensin II antagonists are generally well tolerated with severe side effects occurring very rarely [10]. Despite the estimated 60–80% reduction to CVD risk provided by these preventive medicines [11] non-adherence to long-term medication continues to be a longstanding challenge in healthcare delivery. Evidence from a number of studies have found non-adherence to common CVD medication to be up to 80% for antihypertensives, as high as 75% for statins and up to 29 % for antiplatelets [12–18].

Both international and New Zealand studies have found long-term adherence to statin (a class of lipid-lowering drugs)—e.g. simvastatin, atorvastatin—to be low [12, 13]. In New Zealand, adherence to statin over the 3 years after an Acute Coronary Syndrome (ACS) was found to be 66%, although it was 82% for those on a statin prior to ACS admission [19]. In primary prevention in New Zealand, statin adherence in the first year after initiation of treatment was found to be 63% [20]. A US study found non-adherence to statin to be as high as 56.0% for secondary prevention patients and 56.4% for primary prevention patients [21]. Similarly, a UK based study found patterns of discontinuation of treatment for 41% of patients who are using statin as secondary prevention and 47% of patients who are using statin as primary prevention, although many of these patients restarted their treatment following discontinuation (75% and 72% respectively) [22].

The lack of adherence has dramatic clinical and economic implications. Poor adherence has been associated with approximately twice the risk of death in CVD patients [23, 24]. In the United States, approximately 125,000 deaths, at least 10% of all hospitalisation and significant increase in morbidity are attributed to lack of adherence, annually costing the healthcare system an estimated $100 billion to $289 billion [25]. As such, non-adherence represents a large flaw in current healthcare delivery for chronic condition management. The ability to accurately identify patients at risk of non-adherence could provide a valuable component for clinical decision support to target adherence-promoting interventions.

Patients’ adherence to therapy can be measured in a number of ways; through direct means such as monitoring a drug or its metabolite concentration in blood or urine, or through indirect means including patient self-reporting, the use electronic monitoring devices that record the frequency and time pill bottles have been used, and pill counts [26–28]. A method for measuring adherence that is used with increasing prevalence is by leveraging pharmacy dispense data due to it being non-invasive, low cost and its ability to cover a large population. Although this method makes the assumption that patterns of dispensing are consistent with patterns of actual ingestion/consumption, studies have validated the approach and have shown that this assumption is an acceptable estimate [29, 30]. Adherence measures based on pharmacy dispensing data are numerous; two widely used measures are medication possession ratio (MPR) and proportion of days covered (PDC) [31, 32]. Formally, MPR is defined as

1 $$\begin{aligned} MPR = \left( \frac{\text {Sum of days' supply in period}}{\text {Number of days in period}} \right) \times 100 \end{aligned}$$

and PDC is defined as

2 $$\begin{aligned} PDC = \left( \frac{\text {Number of days in period ``covered''}}{\text {Number of days in period}} \right) \times 100. \end{aligned}$$

**Fig. 1:**
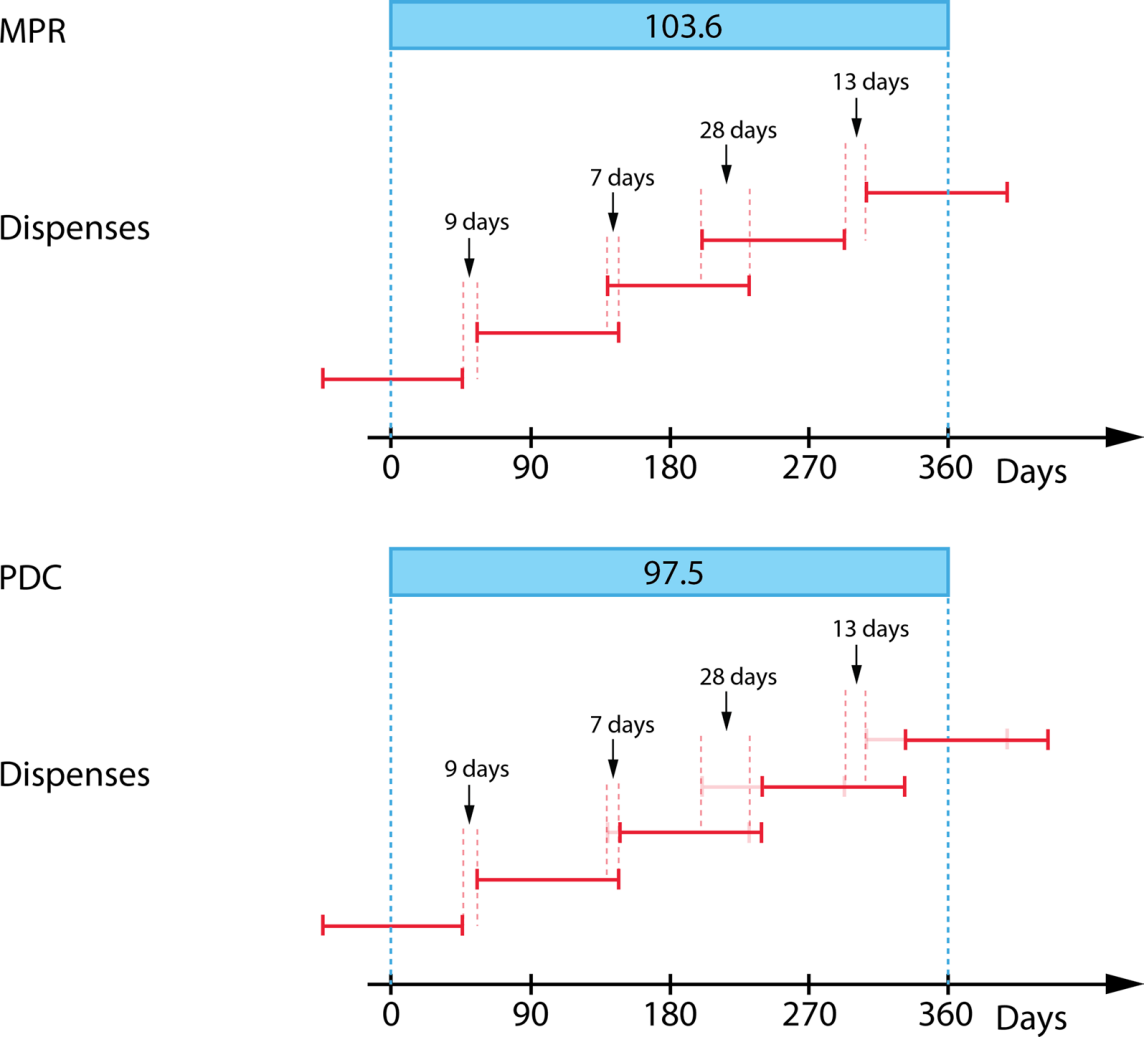
MPR and PDC of two identical dispense patterns. Each dispense represented by the red bar is of 90 days supply, with the left ending of the bar representing the dispense date and the right ending of the bar representing when the supply of the dispense will run out. Top: MPR sums days supply indiscriminately with respect to overlaps and gaps resulting in a value of > 100%. Bottom: PDC accounts for gaps and addresses overlaps by shifting subsequent dispense date to when supply runs out

The notion of “covered” stands for days in the period where the patient can reliably be in possession of their medication. For example, the interval between the dispense date and the number of days supplied after it are considered to be “covered”. Alternatively, if there exists a gap between when the supply runs out and the subsequent dispense this gap will be considered not “covered”. Both measures calculate a percentage value for adherence. Often, the implementation of MPR does not account for gaps, allowing subsequent overlapping dispenses to close earlier gaps leading to overestimation of adherence and potentially a nonsensical value of larger than 100%. PDC is a more conservative measure that accounts for gaps and in addition captures the notion of “stockpile”. Stockpile can happen when there is an overlapping of days supplied between dispenses or when there is leftover medication from the previous periods assessed. For PDC, overlapping subsequent dispenses are shifted allowing succeeding gaps to be covered by supply in the stockpile. PDC avoids the type of overestimation that MPR is prone to and is always $\le$ 100%. Figure 1 illustrates their differences. Here, two identical dispensing patterns are shown where in the case of MPR, the presence of significant overlaps between supplies close off an earlier gap producing an MPR of >100%. Whereas in the case of PDC the earlier gap is maintained through shifting subsequent dispense to the end of the stockpile when overlaps of supplies occur, producing a PDC of 97%.

Logistic regression is a statistical method for modelling the relationship between one or more predictor variables and a dichotomous response variable of the values 1 or 0. It is a function of the odds ratio, and it models the proportion of new incidents developed within a given period of time. Mathematically, the logistic regression model *logit*(*p*) is defined by

3 $$\begin{aligned} \begin{aligned} \frac{p}{1 - p} = \text {exp}(\beta _0 + \beta _1x_1 +\cdots + \beta _{m-1}x_{m-1})\\ \end{aligned} \end{aligned}$$

where $\beta _0$ is the constant and $\beta _1,\ldots ,\beta _{m-1}$ are the coefficients of the predictor variables $x_1,\ldots ,x_{m-1}$, *p* is the probability of the event and $\frac{p}{1-p}$ is the odds for an event [33]. The above equation can also be written as

4 $$\begin{aligned} \text {ln}\left( \frac{p}{1 - p}\right) = \beta _0 + \beta _1x_1 + \cdots + \beta _{m-1}x_{m-1} \end{aligned}$$

Equation 4 is referred to as the logit transformation of the probability of an event, showing that the natural log of the odds ratio is a linear function of predictor variables *x* [33, 34]. The probability of the event *p* can be calculated by [33]

5 $$\begin{aligned} \begin{aligned} p&= \frac{\text {exp}(\beta _0 + \beta _1x_1 +\cdots + \beta _{m-1}x_{m-1})}{1 + \text {exp}(\beta _0 + \beta _1x_1 +\cdots + \beta _{m-1}x_{m-1})}\\&= \frac{\text {exp}({\textbf {x}} \varvec{\beta })}{1 + \text {exp}({\textbf {x}} \varvec{\beta })}\\&= \frac{1}{1 + \text {exp}(-{\textbf {x}} \varvec{\beta })} \end{aligned} \end{aligned}$$

Regression based analysis is widely used in epidemiological studies to uncover the relationship between risk factors and an outcome of interest [35, 36]. In the context of CVD, logistic regression is commonly incorporated in research as a representative traditional statistical method to be compared against more recent methods such as decision tree, gradient boosting as well as less interpretable methods such as SVM, NN, and random forest [37–40]. Researchers have shown the model is competitive and is a valuable tool for risk prediction under certain conditions: moderate sample size ($\sim$ 10,000 patients), small incident rates, and a limited number of predictors [39].

Ridge regression and its classification variant ridge classifier are linear models that address the problem of multicollinearity in the predictor variables [41]. The models are part of a family of penalised regression models including Lasso [42] and Elastic Net [43] that adds a penalty to the loss. This penalty constrains and shrinks the size of the model coefficients, which has a regularisation effect and prevents overfitting. Formally, the ridge regression minimizes

6 $$\begin{aligned} \sum _{i=1}^{n} \left( Y_i - \sum _{j=1}^{p} X_{ij} \beta _{j}\right) ^{2} + L2 \sum _{j=1}^{p} \beta _{j}^{2}. \end{aligned}$$

Here, *n* is the number of samples, *p* is the number of model coefficients, *Y* is the response variable, *X* is the predictor variable and $L2 \ge 0$ is the regularisation parameter. The left hand side of Eq. 6 is the sum of the squared estimate of error and the right hand side of the equation is the penalty term. Ridge regression places a quadratic constraint on $\beta$s, where the regularisation parameter *L*2 controls the amount of shrinkage [44]. *L*2 is a hyperparameter, the value of which needs to be searched, typically through a cross-validation procedure. For classification problems, ridge classifier first modifies the binary response to − 1 and 1 and then treats the task as a regression task, minimising the loss in Eq. 6. The sign of the regressor’s prediction then represents the predicted class.

Ridge regression/classification has shown to be a promising modelling technique in the domain of epidemiology, particularly in high dimensional settings where the number of features is large, such as in genomic data analysis [45, 46]. As a comparatively more interpretable model, it has shown to be competitive against models with the capacity to model non-linear relationships such as Support Vector Machines (SVM) and neural networks (NN) [47].

A multilayer preceptron (MLP) is a densely connected feedforward neural network that in its most simple form consists of 3 layers: input, hidden and output. In contrast to a recurrent neural network, feedforward means information flows from one end of the network to the other without any feedback connections. A feedforward network defines a mapping of $\varvec{y} = f(\varvec{x};\varvec{\theta })$ where $\varvec{x}$ is the input, $\varvec{y}$ the output and $\varvec{\theta }$ the learnt parameters that best approximate the function. Commonly represented as a composite of functions $f(x) = f^{(3)}(f^{(2)}(f^{(1)}(x)))$ where $f^{(i)}$ represents the *i*th layer [48]. For a MLP, $\varvec{\theta }$ consists of the weight matrix $\varvec{W}$ and the bias vector $\varvec{b}$. Mathematically, a 3-layer MLP is defined as

7 $$\begin{aligned}&{\varvec{h}}^{\mathbf{(1)}} = \phi ^{(1)}({\varvec{W}}^{\mathbf{(1)}}\varvec{x} + {\varvec{b}}^{\mathbf{(1)}}) \\&{\varvec{h}}^{\mathbf{(2)}} = \phi ^{(2)}({\varvec{W}}^{\mathbf{(2)}} {\varvec{h}}^{\mathbf{(1)}} + {\varvec{b}}^{\mathbf{(2)}}) \\&\varvec{y} = \phi ^{(3)}({\varvec{W}}^{\mathbf{(3)}}{\varvec{h}}^{\mathbf{(2)}} + {\varvec{b}}^{\mathbf{(3)}}).  \end{aligned}$$

Here, $\varvec{W^{(i)}}$, $\varvec{b^{(i)}}$ and $\phi ^{(i)}$ are the weights, bias and activation for the *i*th layer. $\varvec{h^{(i)}}$ is the output of the layer *i*. The network can be trained end-to-end using backpropagation. Conventionally, with the exception of the output layer, $\phi$ is a non-linear activation, common among which are sigmoid, hyperbolic tangent also known as tanh or the more recently developed rectified linear unit (ReLU). It is the non-linear activation that provides the expressive power of MLP. Even with only a single hidden layer, an MLP can be universal (represent arbitrary functions) under certain technical conditions [49]. Increasing the depth of the network allows the network to represent complex functions more compactly. The hidden layer(s) of MLP can be thought of as learning nonlinear feature mapping, transforming a nonlinearly separable representation of the features to one that is linearly separable [48, 49]. This enables MLP to represent nonlinear relationships, overcoming the limitations of linear models. MLP is often used as an NN comparator among a number of other classification approaches for prediction tasks in the biomedical domain [37–39, 50]. While some studies have shown the strength of MLP over other classical machine learning techniques such as LR, classification and regression tree, gradient boosting, and random forest [37, 38], other studies have shown that MLP is not always superior [39, 50]. Primarily, MLP is known as a model that can capture complex non-linear relationships and interactions among the predictor variables, and is the simplest manifestation of a class of machine learning algorithms (NN). Within the context of this study, the limitation of MLP is that the model is intrinsically non-temporal, and is unable to model temporal relationships and interactions. While MLP is not explicitly a temporal model, it is flexible and will serve as a comparison for the performance of other models.

Recurrent Neural Networks (RNNs) are connectionist models that include edges connecting across adjacent time steps. At time *t* the input to node $h^{(t)}$ is comprised of both the current input data $x^{(t)}$ and the hidden node values from the previous time step $h^{(t-1)}$. At each *t* the hidden node’s value $h^{(t)}$ is then used to calculate the network’s output $\hat{y}^{(t)}$. The forward pass at each time step of a RNN can be fully specified by two equations.

8 $$\begin{aligned} \varvec{h}^{(t)} = \sigma (W^{hx}\varvec{x}^{(t)} + W^{hh}\varvec{h}^{(t-1)} + \varvec{b}^{h} ) \end{aligned}$$

9 $$\begin{aligned} \varvec{\hat{y}}^{(t)} = softmax(W^{yh}\varvec{h}^{(t)} + \varvec{b}^{y}) \end{aligned}$$

where $W^{hx}$ is the weight matrix between the input and the hidden node, $W^{hh}$ is the recurrent weight matrix connecting the hidden layer between adjacent time steps and $W^{yh}$ is the weight matrix between the hidden node and the output. $\varvec{b}^{h}$ and $\varvec{b}^{y}$ are the bias vectors for the hidden node and output respectively. $\sigma$ is a sigmoid activation function and *softmax* is a softmax activation function [51]. Other activation functions may be used, commonly used activations include, ReLU and tanh [52].

RNNs suffer from a problem known as ‘vanishing’ or ‘exploding’ gradient. By unfolding the recurrent edges of the hidden layer, RNNs can be interpreted as deep NNs with one layer per time step where the weights are shared across time steps. The widely used algorithm *backpropagation through time* (BPTT) for training RNNs is the application of backpropagation through the (unfolded) network across time steps. However, as outlined by [53] training RNNs using gradient descend is difficult. The problems arise when the error signal propagated backwards in time vanishes or explodes as the evolution of the backpropagated error exponentially depends on the weights of the recurrent edge. Earlier experiments showed that backpropagation was unable to discover contingencies that span long temporal intervals, settling in suboptimal solutions that learnt short-range dependencies but failed to learn dependencies that are long-range [53].

To address the difficulties in training RNNs, Hochreiter and Schmidhuber introduced *Long Short-Term Memory* (LSTM) [54]. LSTM features a *constant error carrousel* (CEC) to allow constant error to flow through the self-connected units, a multiplicative *input gate unit* and a multiplicative *output gate unit* to protect the network’s memory from perturbation by irrelevant inputs as well as irrelevant memory perturbing other units. The extended unit is called a *memory cell*. The proposed LSTM solved numerous complex tasks requiring the learning of long-range dependencies that were unable to be solved by previous RNN algorithms. Gers et al. further added a *forget gate unit* to LSTM so that it may overcome the weakness of the internal cells’ values growing without bounds when the network is learning from continual input streams that are not previously segmented into training sequences with clearly demarcated beginnings and ends [55]. The forget gates learn to reset the contents of the memory cells once they are no longer needed. Since its introduction, forget gates have proven effective and are now standard in LSTM implementations [51]. The full algorithm of LSTM with forget gate is given by the following equations:

10 $$\begin{aligned}&\varvec{g}^{(t)} = \phi (W^{gx}\varvec{x}^{(t)} + W^{gh}\varvec{h}^{(t-1)} + \varvec{b}^{g}) \end{aligned}$$

11 $$\begin{aligned}&\varvec{i}^{(i)} = \sigma (W^{ix}\varvec{x}^{(t)} + W^{ih}\varvec{h}^{(t-1)} + \varvec{b}^{i}) \end{aligned}$$

12 $$\begin{aligned}&\varvec{f}^{(t)} = \sigma (W^{fx}\varvec{x}^{(t)} + W^{fh}\varvec{h}^{(t-1)} + \varvec{b}^{f}) \end{aligned}$$

13 $$\begin{aligned}&\varvec{o}^{(t)} = \sigma (W^{ox}\varvec{x}^{(t)} + W^{oh}\varvec{h}^{(t-1)} + \varvec{b}^{o}) \end{aligned}$$

14 $$\begin{aligned}&\varvec{s}^{(t)} = \varvec{g}^{(t)} \odot \varvec{i}^{(t)} + \varvec{s}^{(t-1)} \odot \varvec{f}^{(t)} \end{aligned}$$

15 $$\begin{aligned}&\varvec{h}^{(t)} = \phi (\varvec{s}^{(t)}) \odot \varvec{o}^{(t)} \end{aligned}$$

Here $\varvec{g}^{(t)}$ is the input node that takes activation from the input layer $\varvec{x}^{(t)}$ and the hidden layer $\varvec{h}^{(t-1)}$. The superscripts *t* and $t-1$ indicate time steps, where *t* is the current time step and $t-1$ the previous time step. $W^{gx}$ and $W^{gh}$ are weights for the input layer to the input node and the hidden layer to the input node respectively. $\phi$ is a *tanh* activation function for the summed weighted input and bias vector $\varvec{b}^{g}$. $\varvec{i}^{(t)}$, $\varvec{f}^{(t)}$ and $\varvec{o}^{(t)}$ are the input, forget and output gate units. Each gate also takes activation from the summed weighted $\varvec{x}^{(t)}$, $\varvec{h}^{(t-1)}$ and their respective bias vectors. Here, $\sigma$ is a *sigmoid* function; if the value of the gate is one, all flow is passed through, if the value is zero, the flow is entirely blocked. $\varvec{s}^{(t)}$ is the internal state of the memory cell. $\odot$ denotes pointwise multiplication. The value of the internal state is updated by the sum of the input node $\varvec{g}^{(t)}$ multiplied by the input gate unit $\varvec{i}^{(t)}$ and the internal state from the previous time step $\varvec{s}^{(t-1)}$ multiplied by the forget gate unit $\varvec{f}^{(t)}$. The value of the hidden layer $\varvec{h}^{(t)}$ is then derived first by passing the internal state $\varvec{s}^{(t)}$ through a *tanh* function and then by multiplying the value of the output gate unit $\varvec{o}^{(t)}$.

Recently, a number of researchers have applied RNN, in particular, LSTM in the biomedical domain. The task of making predictions in the healthcare domain is influenced by a recency effect akin to human memory as well as simultaneously requiring learning the dependencies of distant past events, for these reasons the architecture of LSTM is well suited. These studies include [56] where LSTM is used to model multivariate pediatric intensive care time series to predict diagnoses and [57] where LSTM is used to jointly analyse episodic clinical events and continuous monitoring data in the ICU settings to predict deterioration of patient conditions and their length of stay.

Pham et al. [58] proposed an extension to the LSTM model called DeepCare. The model regulates the input gate of LSTM with information on the patient’s diagnoses and admission method (planned or unplanned) and regulates the forget and output gates with information about the patient’s diagnoses and procedures or medications received. DeepCare not only was able to learn long term dependencies in a patient’s disease trajectory, it can learn the confounding interaction between disease progression and treatment. The authors demonstrated the superiority of DeepCare at the task of diagnosis and intervention prediction against a Markov model and a plain RNN for two distinct diseases; diabetes and mental health. In the same study, DeepCare was shown to be superior to Support Vector Machine (SVM) and Random Forest in predicting future risk of readmissions.

In one particular recent study LSTM was applied to the task of medication adherence prediction. In this study, patients who were on self-administered injection therapy were monitored through a internet of things (IoT)-connected smart sharps bin. Data collected from the smart bin was then used to develop ensemble and deep learning machine learning models to predict patient adherence at the next scheduled injection [59]. Against model comparators including: extreme gradient boosting, extremely randomized trees, random forest, gradient tree boosting, MLP, and RNN; LSTM was found to be the best performing model on the held-out test set, achieving AUC of 0.8902 [59].

Similar to the above mentioned study, other recent studies in medication adherence have also underscored the temporal patterns present in patient adherence behaviour and the relationships between clinical events in patients history to adherence [60, 61]. To the best of our knowledge, the prediction of long-term medication adherence using temporal deep learning models on a large routinely collected population data set has not been investigated. The current study integrates patient history into the analytics task and aims to identify individuals within a population who might be at risk of medication non-adherence. The experiment focuses on lipid-lowering pharmacological treatment. All patients included in the cohort of the study have had at least one dispense of lipid-lowering medication in the observation window, with a two week look ahead past the index date. Informed by clinical practice, the study makes the assumption that once the patient has been prescribed a lipid-lowering medication, the patient should continue to be prescribed lipid-lowering medication; thus the lack of dispensed medication supply indicates a flaw in the CVD risk management process, such as failure to get repeat prescriptions or failure to have them dispensed.

Given the prolonged nature of pharmacological therapy for CVD, the hypothesis for this study is that computational methods that allow the integration of patient history including the history of pharmaceutical dispensing, hospitalisation, and lab test results (indicating patients’ physiological changes), through temporal modelling will aid predictive performance. The study investigates explicitly temporal models for sequential modelling including Long short-term memory (LSTM) recurrent neural network (RNN) architecture and simple recurrent neural networks (Simple RNN) as well as non-temporal models multilayer perceptron (MLP), ridge classifier (RC) and logistic regression (LR). LSTM contains internal mechanisms that facilitate the learning of important events and discarding of unimportant events in the distant past, overcoming the problem known as vanishing and exploding gradient suffered by Simple RNN [51, 54]. For this prediction task, the hypothesis that by lengthening the observation window temporal models, specifically LSTM, might gain a performance advantage is also investigated.

## Methods

### Adherence measure and medication switching

**Fig. 2:**
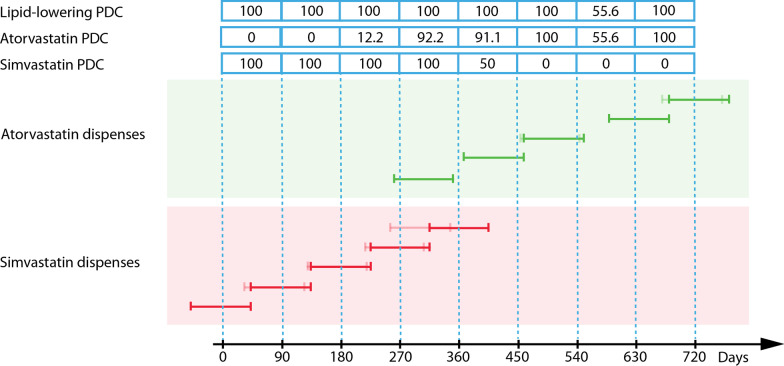
Dispensing pattern of simvastatin to atorvastatin switching and their respective PDC measures. A Lipid-lowering PDC is calculated by summing their respective PDC and setting an upper bound of 100

The current study uses PDC as the measure of adherence, but the commonly-observed phenomenon of medication switching in long-term therapy must be addressed. A frequent example in CVD management in New Zealand is the switching from simvastatin to atorvastatin. These two drugs constitute the majority of lipid-lowering medication prescribed in the country. Atorvastatin is a more potent drug at blocking target enzyme HMGCoA [62] and prior to September 2010 approval for atorvastatin required first-line use of simvastatin [63]. During medication switching, the total days “covered” of a class of drugs could exceed 100% even if one uses PDC as the measure. To apply the method of shifting the dispense date based on overlap across the two drugs is unrealistic as once the new drug is dispensed, the likely scenario is the patient will discontinue taking the older medication. In this study this issue is addressed by summing the PDC across any lipid-lowering drugs dispensed in the same quarter and set an upper-bound for the value to 100%. Figure 2 illustrates the method using simvastatin to atorvastatin switching as an example.

### Data sources and cohort

PREDICT is a web-based CVD risk assessment and management decision support system developed for primary care in New Zealand. The system is integrated with the general practice electronic health record (EHR) and since its deployment in 2002 has produced a constantly growing cohort of CVD risk profiles. Through the use of encrypted National Health Identifier number (NHI), the de-identified cohort is annually linked to other routinely collected databases to produce a research cohort. The PREDICT cohort and its use in improving CVD risk assessment has been described in detail previously [64, 65]. Vascular Informatics Using Epidemiology and the Web (VIEW) is a research programme with the goal of reducing inequities in vascular disease outcomes. The VIEW team has made a de-identified extract from the PREDICT cohort and linked data available for the present study [66].

The current study links the PREDICT cohort to TestSafe (Auckland regional laboratory test results [67]) and national collections by the Ministry of Health - the Pharmaceutical collection, the National Minimum Dataset (hospital events) and the Mortality Collection [68]. TestSafe is used to obtain lab test results of clinically relevant measures: high-density lipoproteins (HDL), low-density lipoproteins (LDL), triglyceride (TRI), total cholesterol (TCL), total cholesterol to high density lipoprotein ratio (TC/HDL), glycated hemoglobin (HbA1c) and estimated Glomerular Filtration Rate (eGFR). The Pharmaceutical collection is used to obtain dispensing history of medication relevant to the management of CVD including lipid-lowering, blood pressure lowering, antiplatelets, and anticoagulants as well as dispensings of drugs used in the management of important comorbidities e.g. insulin. The National Minimum Dataset (NMDS) is used to identify hospitalisation with their dates of admission and discharge and diagnosis. The Mortality collection enables the identification of patients who died during the study period and their cause of death. From these sources, history of CVD, treatment trajectories, important comorbidities as well as CVD events can be derived.

**Table 1:** VIEW CVD categories: CVD history, CVD mortality and CVD outcome, feature names under the categories and feature descriptions

Category	Feature name	Description
*VIEW CVD categories*		
History	HX_BROAD_CVD	History of broad CVD
	HX_ATHERO_CVD	History of atherosclerotic CVD
	HX_CHD_DIAGS	History of coronary heart disease (diagnoses)
	HX_ACS	History of acute coronary syndrome
	HX_MI	History of myocardial infarction
	HX_UNST_ANGINA	History of unstable angina
	HX_ANGINA	History of angina
	HX_OTHER_CHD	History of other coronary disease
	HX_CHD_PROCS	History of coronary heart disease
	HX_PCI	History of percutaneous coronary intervention
	HX_CABG	History of coronary artery bypass graft
	HX_OTHER_CHD_PROCS	History of other coronary procedure
	HX_PVD_DIAGS	History of peripheral vascular disease
	HX_PVD_PROCS	History of peripheral vascular procedure
	HX_HAEMORRHAGIC_STROKE	History of haemorrhagic stroke
	HX_CEVD	History of cerebral vascular disease
	HX_ISCHAEMIC_STROKE	History of ischaemic stroke
	HX_TIA	History of transient ischaemic attack
	HX_OTHER_CEVD	History of other cerebral vascular disease
	HX_HEART_FAILURE	History of heart failure
	HX_ATRIAL_FIBRILLATION	History of atrial fibrillation
Mortality	MORTALITY_BROAD_CVD_WITH	Death involving broad CVD
	_OTHER	
	MORTALITY_OTHER_RELATED	Death involving other related CVD
	_CVD_DEATHS	
Outcome	OUT_BROAD_CVD	Outcome of broad CVD
	out_athero_cvd	Outcome of atherosclerotic CVD
	OUT_CHD	Outcome of coronary heart disease
	OUT_MI	Outcome of myocardial infarction
	OUT_ACS	Outcome of acute coronary syndrome
	OUT_UNST_ANGINA	Outcome of unstable angina
	OUT_ANGINA	Outcome of angina
	OUT_OTHER_CHD	Outcome of acute coronary syndrome
	OUT_PVD_DIAGS	Outcome of peripheral vascular disease
	OUT_PVD_PROCS	Outcome of peripheral vascular procedure
	OUT_PCI_CABG	Outcome of percutaneous coronary intervention
	OUT_HAEMORRHAGIC_STROKE	Outcome of haemorrhagic stroke
	OUT_CEVD	Outcome of cerebral vascular disease
	OUT_ISCHAEMIC_STROKE	Outcome of ischaemic stroke
	OUT_TIA	Outcome of transient ischaemic attack
	OUT_OTHER_CEVD	Outcome of other cerebral vascular disease
	OUT_HEART_FAILURE	Outcome of heart failure
	OUT_ATRIAL_FIBRILLATION	Outcome of atrial fibrillation

Feature names prefixed with MORTALITY or OUT are used to identify outcome events (with the exception of OUT_ATRIAL_FIBRILLATION)

A lookup table constructed by the VIEW research team is used to identify relevant chemical names from the Pharmaceutical collection. Identified chemical names using this lookup table are grouped into 3 broad categories Lipid-lowering, CVD and Other. Similarly, a lookup table constructed by the VIEW research team is used to identify ICD-10 codes in the hospitalisation collection that are related to CVD conditions: more specifically, International Statistical Classification of Diseases and Related Health Problems, Tenth Revision, Australian Modification, ICD-10-AM, which was used in New Zealand from 1999 to 2019 [69]. The conditions are broadly in two categories: history and outcome, with the addition of mortality. For the list of the CVD conditions and their respective categories see Table 1. For the definitions of listed conditions see https://wiki.auckland.ac.nz/display/VIEW/Complete+Variable+Names+Index

**Fig. 3:**
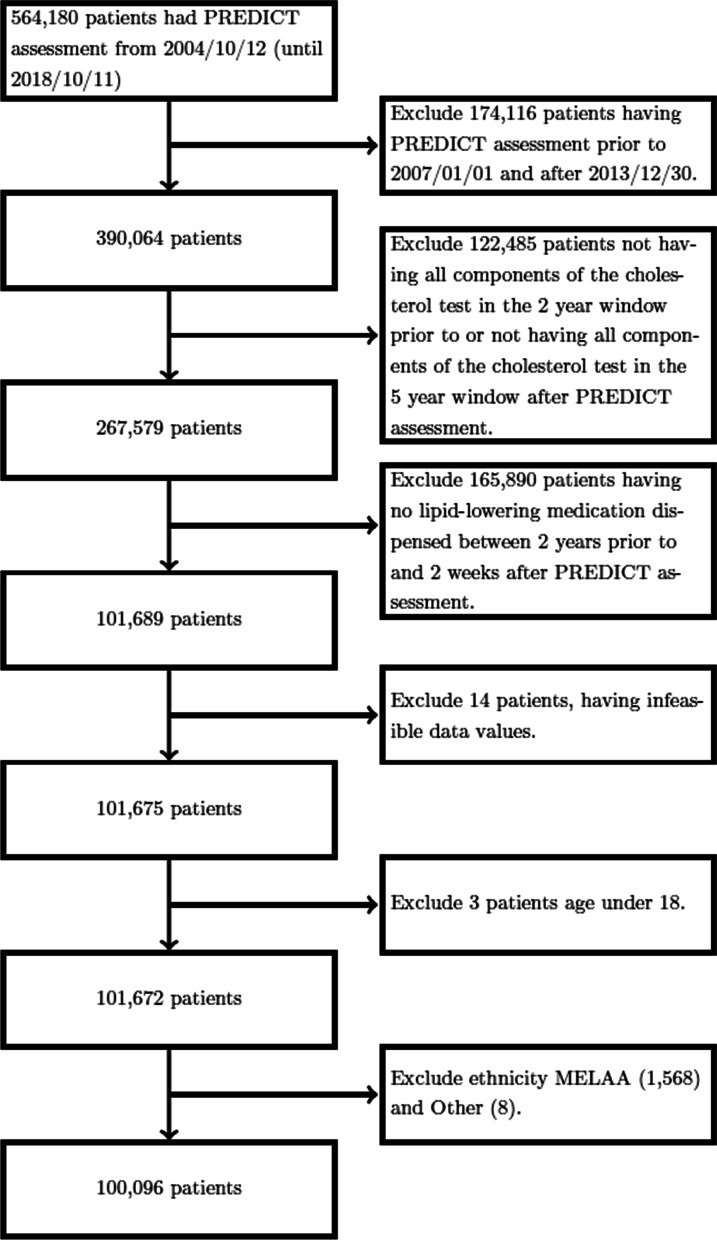
Flow chart of study cohort selection

The study cohort was selected through a number of exclusion criteria. First, patients having their PREDICT assessment prior to 01/01/2007 and after 30/12/2013 are excluded as their pharmaceutical records are censored in the observation or prediction windows. Second, informed by our interest in integrating the temporal pattern of disease states, patients without all components of cholesterol test results (HDL, LDL, TRI, TCL and TC/HDL) reported in either the observation or prediction windows are excluded. Third, informed by our interest in integrating the temporal pattern of disease management process, patients without lipid-lowering medication dispensed in the observation window with a 2-week look ahead post PREDICT assessment (to account for patients prescribed lipid-lowering medication in response to the PREDICT assessment) are excluded. Patients with infeasible data values and patients under the age of 18 are excluded. Lastly, Ethnicity MELAA (Middle Eastern, Latin American and African; which comprises only 1.5% of the New Zealand population [70]) and Other are excluded due to small sample size. See Fig. 3 for the study cohort selection flowchart.

Of the 100,096 patients in the selected cohort, 25,419 patients have prior history of CVD, defined as having a hospital admission prior to their PREDICT assessment date with an ICD-10-AM code matching the ‘broad CVD history’ category (HX_BROAD_CVD) defined by VIEW.

### Study design

**Fig. 4:**
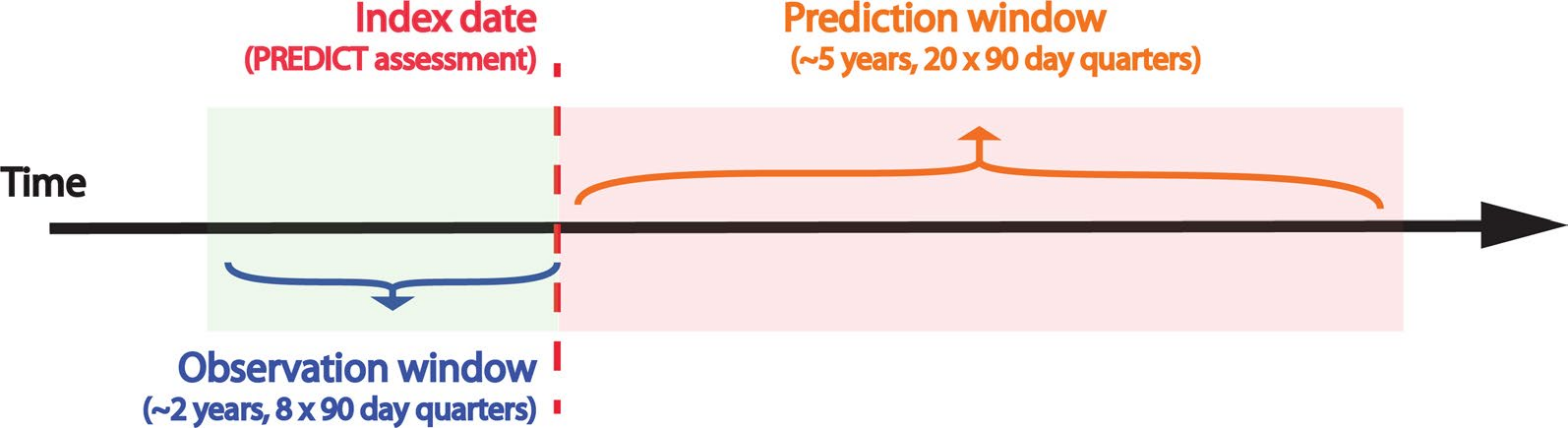
Study design showing date range from index date for the observation window (shaded in green) and the prediction window (shaded in red)

A study design is formulated using each patient’s PREDICT assessment as the index date, and the $\sim$ 2 years (8 $\times$ 90 day quarters) prior to the index date and the $\sim$ 5 years (20 $\times$ 90 day quarters) after the index date as the observation window and the prediction window respectively. (see Fig. 4). A time-step of a quarter (90 days) is used for the constructed time-series. This decision is informed by 90 days being the most common value in the pharmaceutical record for DAYS_SUPPLY of Lipid-lowering medication, the CVD preventive treatment of principal interest. A $\sim$ 5 years interval for the prediction window is chosen because it aligns with MoH guildlines for CVD risk assessment and is underpinned by the fact that patients’ CVD risk and risk management can change considerably over a longer period (i.e. 10 years), most randomised controlled trials of CVD medications are based on a period of 5 years or less and that practitioners are accustomed to this approach [3]. A $\sim$ 2 years interval for the observation window is chosen in the interest of retaining enough samples in the data set, as dispense data extracted from the pharmaceutical collection begins from 2005, the longer the observation window grows the larger the number of samples that will need to be excluded.

### Descriptive statistics

Based on the study design outlined in the section Study design and the result of the cohort selection outlined in the section Data sources and cohort, quarterly time-series based on 90-day quarters are constructed for each patient in the cohort using the linked data outlined in the section Data sources and cohort. The features of the data fall into 8 categories: Demographic, Lipids, Lipid-lowering drugs, CVD drugs, Other drugs, Hospitalisation, HbA1c and eGFR, and PREDICT. See Tables 2, 3, 4, 5 and 6 for the features’ descriptive statistics, and Table 7 for the variable descriptions of the PREDICT variables.

**Table 2:** Descriptive statistics: demographic variables

ID	100096
Sex	
Male	56,557 (56.5%)
Female	43,539 (43.5%)
AGE (at index date)	
Mean (SD)	61.82 (11.29)
18–24	48
25–34	691
35–44	5690
45–54	20,380
55–64	32,885
65–74	28,261
75–84	10,379
85+	1762
NZDEP	
1	21,167
2	19,074
3	17,141
4	18,903
5	23,811
Ethnicity	
European	56,641
M$\bar{a}$ori	9977
Pacific	14,878
Chinese/other Asian	8971
Indian	9629
DIED (%)	6634 (6.6%)

Number of patients in each category

**Table 3:** Descriptive statistics: cholesterols

TEST (%)	885936 (31.6%)
HDL mean (SD)	1.28 (0.37)
LDL mean (SD)	2.62 (0.96)
TRI mean (SD)	1.74 (1.04)
TCL mean (SD)	4.69 (1.13)
TC/HDL mean (SD)	3.85 (1.15)
TESTED (%)	2,698,599 (96.3%)

TEST and TESTED are auxiliary binary features indicating whether the patient had a cholesterol test in this quarter (encompassing HDL, LDL, TRI, TCL and TC/HDL), and whether the patient has ever had a cholesterol test respectively. If the patient has any of the listed elements tested in a quarter the value of TEST will be 1, otherwise 0. In a patient’s time-series, the value of TESTED will be 0 prior to the patient having their first cholesterol test. Once the patient has had a cholesterol test, the value of TESTED will switch to 1 and stay at 1 for the remainder of the time-series. The statistics for TEST and TESTED are the number of quarters in the entire data set where the feature contained a 1 and its relative percentage

**Table 4:** Descriptive statistics: hospitalisation

NUMBER_OF_DAYS > 0	
Mean (SD)	6.37 (11.87)
ACUTE_ADM	54,448
HX_BROAD_CVD	32,542
HX_ATHERO_CVD	30,259
HX_CHD_DIAGS	23,207
HX_ACS	16,777
HX_MI	13,799
HX_UNST_ANGINA	6596
HX_ANGINA	8489
HX_OTHER_CHD	20,416
HX_CHD_PROCS	12,771
HX_PCI	8646
HX_CABG	5659
HX_OTHER_CHD_PROCS	335
HX_PVD_DIAGS	5301
HX_PVD_PROCS	3551
HX_HAEMORRHAGIC_STROKE	1204
HX_CEVD	8403
HX_ISCHAEMIC_STROKE	5878
HX_TIA	3159
HX_OTHER_CEVD	772
HX_HEART_FAILURE	8079
HX_ATRIAL_FIBRILLATION	10,902
MORTALITY_BROAD_CVD_WITH_OTHER	17,463
MORTALITY_OTHER_RELATED_CVD_DEATHS	2416
OUT_BROAD_CVD	16,421
OUT_ATHERO_CVD	14,308
OUT_CHD	9689
OUT_MI	5944
OUT_ACS	7445
OUT_UNST_ANGINA	2104
OUT_ANGINA	3300
OUT_OTHER_CHD	3539
OUT_PVD_DIAGS	1537
OUT_PVD_PROCS	1922
OUT_PCI_CABG	5758
OUT_HAEMORRHAGIC_STROKE	521
OUT_CEVD	4364
OUT_ISCHAEMIC_STROKE	3011
OUT_TIA	1598
OUT_OTHER_CEVD	50
OUT_HEART_FAILURE	3096
OUT_ATRIAL_FIBRILLATION	3288

Number of patients who had acute hospital admission within their time-series and number of patients who had hospitalisations with clinical code mapping to the specified category in their time-series. See Table 1 for the descriptions of the features

**Table 5:** Descriptive statistics: HbA1c and eGFR

HBA1C mean (SD)	47.98 (15.20)
TEST_HBA1C	810,747 (28.9%)
TESTED_HBA1C	2,268,295 (80.9%)
EGFR mean (SD)	77.85 (20.11)
TEST_EGFR	1,041,487 (37.2%)
TESTED_EGFR	2,694,767 (96.1%)

TEST_HBA1C, TESTED_HBA1C, TEST_EGFR and TESTED_EGFR are auxiliary binary features indicating whether the patient had a HbA1c or a serum creatinine (from which eGFR is derived) test in this quarter, and whether the patient has ever had a HbA1c and serum creatinine test respectively. If the patient has a HbA1c or serum creatinine test in a quarter the value of their respective TEST_HBA1C or TEST_EGFR will be 1, otherwise 0. In a patient’s time-series, the value of TESTED will be 0 prior to the patient having their first HbA1c or serum creatinine test. Once the patient has had a HbA1c or serum creatinine test, the value of their respective TESTED_HbA1c or TESTED_EGFR will switch to 1 and stay at 1 for the remainder of the time-series. the statistics are the number of quarters in the entire data set where the feature contained a 1 and its relative percentage

**Table 6:** Descriptive statistics: PREDICT

PT_SBP mean (SD)	132.25 (16.99)	PT_GEN_LIPID	
PT_SBP2 mean (SD)	132.57 (17.24)	0 (None)	92,492
PT_DBP mean (SD)	78.70 (10.25)	1 (Familial hypercholesterolaemia)	5569
PT_DBP2 mean (SD)	79.07 (10.30)	2 (Familial defective apoB)	20
PT_SMOKING		3 (Familial combined dyslipidaemia)	499
0 (Never)	66,896	4 (Other genetic lipid disorder)	1516
1 (Quit>12months)	20,162	PT_RENAL	
2 (Quit$\le$12months)	1901	0 (Missing value)	64,131
3 (Up to 10/day)	6249	1 (No nephropathy)	27,585
4 (11-19/day)	3046	2 (Confirmed microalbuminuria)	5996
5 (20+/day)	1842	3 (Overt diabetic nephropathy)	1975
PT_EN_TCHDL mean (SD)	3.90 (1.22)	4 (Non-diabetic nephropathy)	409
PT_DIABETES		PT_DIABETES_YR mean (SD)	8.19 (7.30)
0 (No diabetes)	64,125	PT_ATRIAL_FIBRILLATION	
1 (Type 1)	1267	0 (Missing value)	21
2 (Type 2)	32,754	1 (None)	95,292
3 (Type unknown)	1950	2 (Confirmed atrial fibrilation)	4783
PT_FAMILY_HISTORY	20,162	PT_IMP_FATAL_CVD*	2998

PT_SMOKING, PT_DIABETES, PT_FAMILY_HISTORY, PT_GEN_LIPID, PT_RENAL, PT_ATRIAL_FIBRILLATION and PT_IMP_FATAL_CVD show number of patients in each category. See Table 7 for the variable descriptions

**Table 7:** PREDICT variables and their descriptions

Variable name	Description
PT_SBP	Current systolic blood pressure (sitting)
PT_SBP2	Previous systolic blood pressure (sitting)
PT_DBP	Current diastolic blood pressure (sitting)
PT_DBP2	Previous diastolic blood pressure (sitting)
PT_SMOKING	Smoking history or current status
PT_EN_TCHDL	TC/HDL cholesterol result
PT_DIABETES	Diabetes status
PT_FAMILY_HISTORY	Family history of premature CVD
PT_GEN_LIPID	Diagnosed Genetic Lipid Disorder
PT_RENAL	Renal disease status
PT_DIABETES_YR	Number of years since diabetes diagnosis
PT_PT_ATRIAL_FIBRILLATION	ECG confirmed Atrial Fibrillation
PT_IMP_FATAL_CVD*	Improved fatal CVD using mortality record and 28 day rule

*This feature captures all patients with CVD as cause of death on their death certificate with or without hospitalisation. In addition, those without CVD recorded on their death certificate but who had a CVD hospital admission up to 28 days before their date of death are included. The VIEW research group refers to this as the “the 28 day rule” for reclassifying non-CVD death as CVD death

### Prediction outcome

The adherence prediction problem is formulated as a binary classification task: predicting adherent or non-adherent. The feature LL_PDC is a time-series that sums over all lipid-lowering PDCs: simvastatin, bezafibrate, atorvastatin, ezetimibe, nicotinic acid, acipimox, cholestyramine, cholestipol hydrochloride, pravastatin, ezetimibe with simvastatin and gemfibrozil, with an upper bound of 100 as described in the section Adherence measure and medication switching. Thus, any sums of lipid-lowering PDCs exceeding the value of 100 are set to 100. This feature is used to assess patient adherence using a historical and widely applied threshold of $\ge 80$ indicating adherence [71, 72]. Here, patients’ mean LL_PDC in the prediction window determines their class ($\ge 80$ equals class label 1 for adherence, 0 for non-adherence otherwise). Of the 100,096 patients included in the study cohort, 42,428 patients are classed as non-adherent and 57,668 patients are classed as adherent.

### Prediction models and evaluation

The models LSTM, Simple RNN, MLP, RC and LR are compared. The input data for LSTM and Simple RNN are explicitly sequential, and the input data for MLP, RC as well as LR are flattened across the time-step dimension and concatenated. To examine the effect of multicollinearity as well as the effect of using history on RC and LR, two other input data sets are constructed. First, instead of concatenating the features across multiple time-steps, an input data set is constructed that uses the values of the last time-step in the observation window (quarter 8) for features that are invariable across time (i.e. SEX, ETHNICITY, NZDEP) and the mean value of features that are variable across time (i.e. TC/HDL, LL_SIMVASTATIN, HX_BROAD_CVD). Here, an exception is AGE where the value at the 8th quarter is used. This data set is from here on referred to as *aggregated*. Second, an input data set is constructed using only the values of the last quarter in the observation window. This data set is from here on referred to as *last quarter*.

**Fig. 5:**
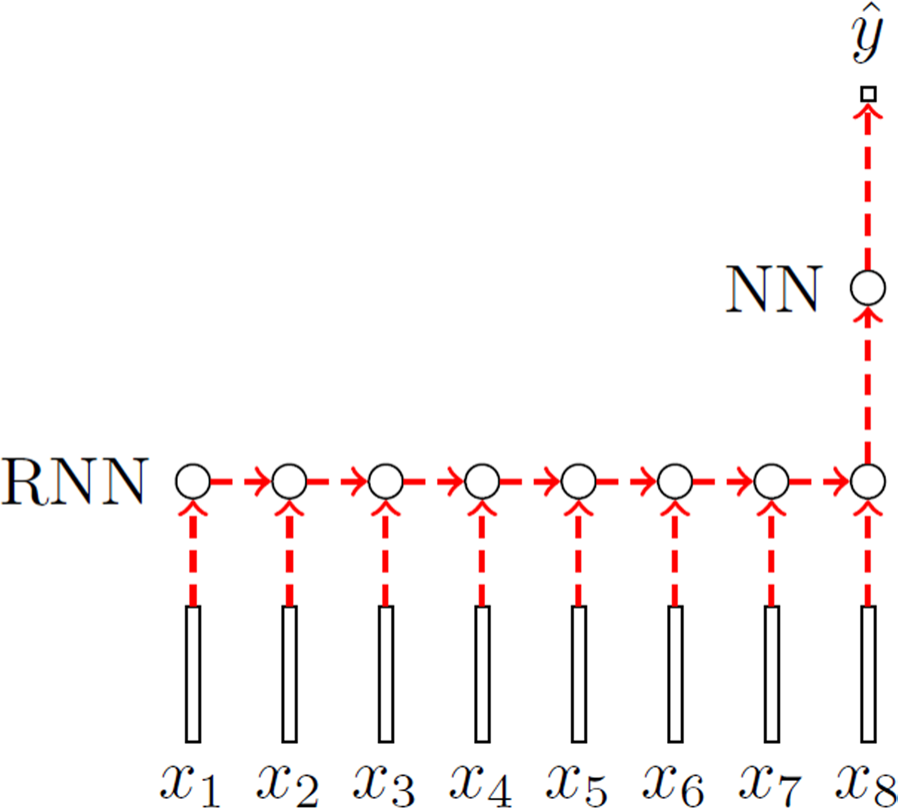
An unrolled view of RNN across the time-step dimension. Here, RNN can be a layer of Simple RNN or LSTM. NN is a layer of densely connected NN with softmax activation. $x_{n}$ are the inputs across *n* time-steps. $\hat{y}$ is the output

All NN models are connected to a 2-unit densely connected output layer. This layer uses the softmax activation from which the probability distribution of the two classes is derived. The RNN models (LSTM and Simple RNN) require an architecture that takes multiple inputs across the observation window and only outputs once at the last time-step. The unrolled view across the time-step dimension of the RNN models is shown in Fig. 5.

### Software setup

Experiments are carried out using Python 3.6.8 [73], with neural network models using library Keras 2.2.4 [52] with Tensorflow 1.13.1 [74] backend and linear models RC and LR using library Scikit-learn 0.21.2 [75]. Experiments also used R version 3.6.0, package pROC 1.16.2 [76] for conducting DeLong’s test and SciPy library 1.5.4 for conducting Kolmogorov–Smirnov test [77].

### Procedures for hyperparameter search

**Fig. 6:**
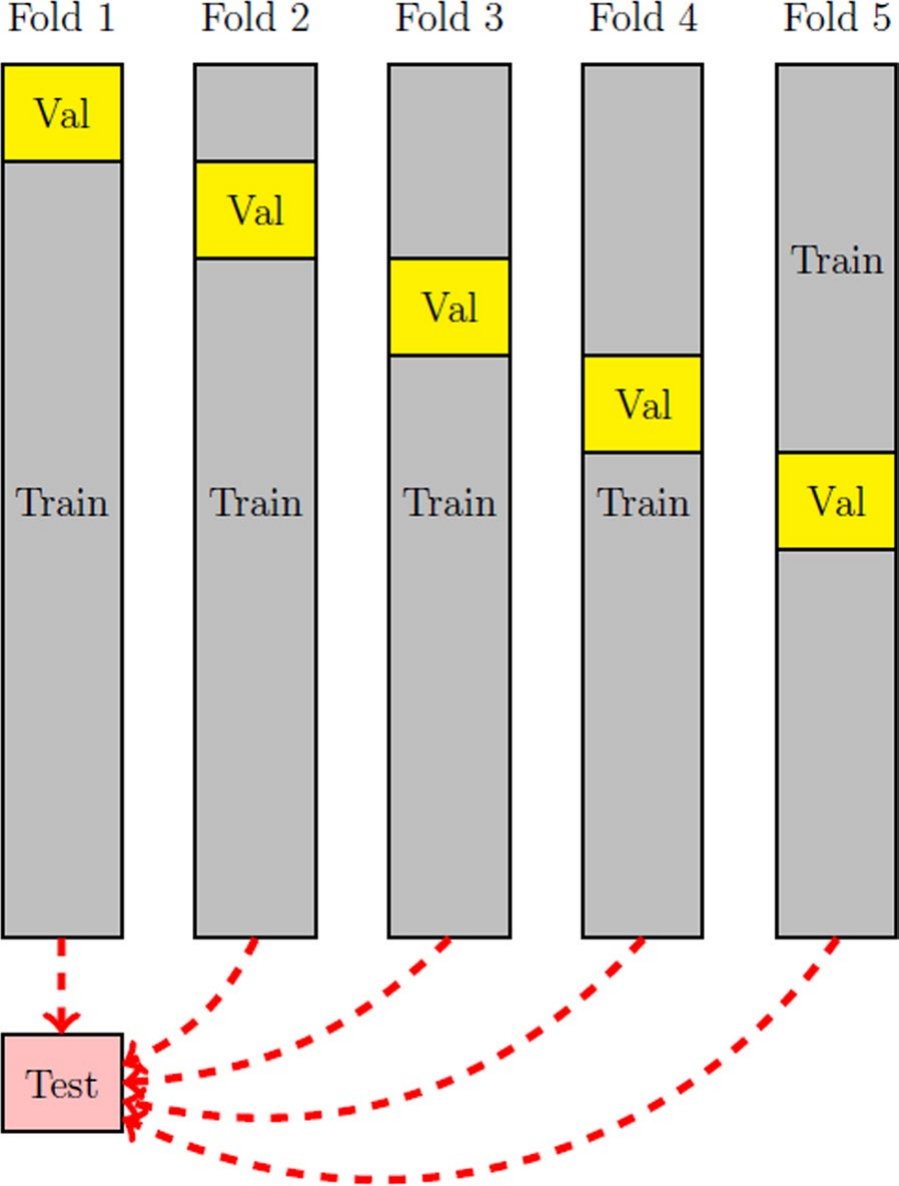
Illustration of the procedure used in splitting data into test, train and validation sets across different folds

This section outlines the procedures carried out to search for the optimal set of hyperparameters for the LSTM, Simple RNN and MLP models. The samples in the data set are first randomly shuffled, and then from the entire data set, 10,096 samples are set aside as the test set and removed from the search process. The remaining data (90,000 samples) are used in the search process. For each combination of hyperparameters, a fivefold cross validation is carried out where while the proportion of data used for the train and validation sets are consistent, with 90% train (81,000 samples) and 10% validation (9000 samples), different splits of train and validation sets are used in the experiments. See Fig. 6 for a visual illustration of how the data is split into train and validation sets across the 5 folds. In these experiments, the validation error is monitored and the lowest mean validation error is used to determine the best set of hyperparameters.

For all experiments, the optimizer ADAM [78] is used due to its capacity to adaptively adjust the learning rate during the training process and because its default hyperparameters have been shown to work on a range of problems. The ADAM optimizer is used with the default hyperparameter values outlined in [78]. These hyperparameter values are, learning rate $\alpha = 0.001$, the exponential decay rate for the 1st moment estimate $\beta _1 = 0.9$, the exponential decay rate for the 2nd moment estimate $\beta _2 = 0.999$, and the small constant for numeric stability $\hat{\epsilon } = 1e-7$ [52]. See Table 8 for the found optimal hyperparameters of the NN models.

**Table 8:** NN model hyperparameters for the adherence prediction experiment

Models	Hyperparameters
LSTM	Layers: 1 LSTM and 1 Dense
	Units: 4 (LSTM) and 2 (Dense)
	Batch size: 1024
	L2: 9.261e−3
	Loss: categorical cross-entropy
	Epochs: 100
Simple RNN	Layers: 1 Simple RNN and 1 Dense
	Units: 8 (Simple RNN) and 2 (Dense)
	Batch size: 4096
	L2: 1.202e−2
	Loss: categorical cross-entropy
	Epochs: 100
MLP	Layers: 3 Dense and 2 Dropout
	Units: 64, 64 and 2
	Batch size: 256
	Dropout rate: Layer 1 2.152e−1
	Layer 2 1.758e−1
	Loss: categorical cross-entropy
	Epochs: 50

Scikit-learn’s RidgeClassifierCV class [75] provides an implementation of ridge classifier that uses a built-in generalised cross-validation to search for the optimal *L*2 value from an array of values. For this experiment, the values $1e^{-6}$, $1e^{-5}$, $1e^{-4}$, $1e^{-3}$, $1e^{-2}$, 0.1, 1 and 10.0 were searched. See Table 9 for the found optimal *L*2 values and their respective accuracy on the validation set.

**Table 9:** Optimal *L*2 values found for RidgeClassifier for adherence prediction and their respective accuracy on the validation set

	*L*2	Accuracy
RC	0.1	0.736
RC (aggregated)	0.01	0.713
RC (last quarter)	0.01	0.726

### Assessment of model performance

Once the optimal hyperparameters for each NN model have been found, the models are trained using the found hyperparameters with the data split shown in Fold 1 in Fig. 6. The test set that is held aside is then used to assess model performance. The linear models RC, LR and their respective *aggregated* and *last quarter* variants are trained using the same training samples in Fold 1 and use the same test samples to measure model performance. For RC, the value of *L*2 is estimated using the training samples, accuracy reported are calculated using the validation set and model performance is assessed based on prediction made using the test set.

The performance of the models on the test set are compared by using the metric of receiver operating characteristic (ROC) area under the curve (AUC) score [75, 79]. To assess the statistical significance of the difference in performance between the models, DeLong’s test is used to conduct pairwise comparisons. DeLong’s test is a nonparametric test that can be used to obtain a p-value when two ROC AUC are compared [80]. The Bonferroni adjustment is used to address the increased likelihood of making a type I error when making multiple comparisons [81]. Sensitivity analysis is conducted by randomly resampling the test set with replacement. Here, 1000 (repeats) of 10,096 (resampled test sets) are used to assess model performance, with each model producing 1000 ROC AUC scores. The models’ scores are then compared using two-sample Kolmogorov-Smirnov test [77, 82].

A potentially important consideration for adherence analysis using dispensing data is the number of days the patient is hospitalised in the prediction window. During this time the patient is given their medication but is not recorded as part of the dispensing data (as inpatient medicine supply is not tracked within the community-pharmacy based data source). However, in the extracted de-identified quarterly time-series it was difficult to accurately track the number of days the patient is an inpatient as, for instance, consecutive days of outpatient visits and a single inpatient stay could appear identical. It was determined that only 0.54% of the training set changed from non-adherent to adherent with a maximal estimate of in-patient hospitalisation days, and therefore hospitalisation was ignored in computing PDC in the observation period.

### Post-PREDICT experiment

**Fig. 7:**
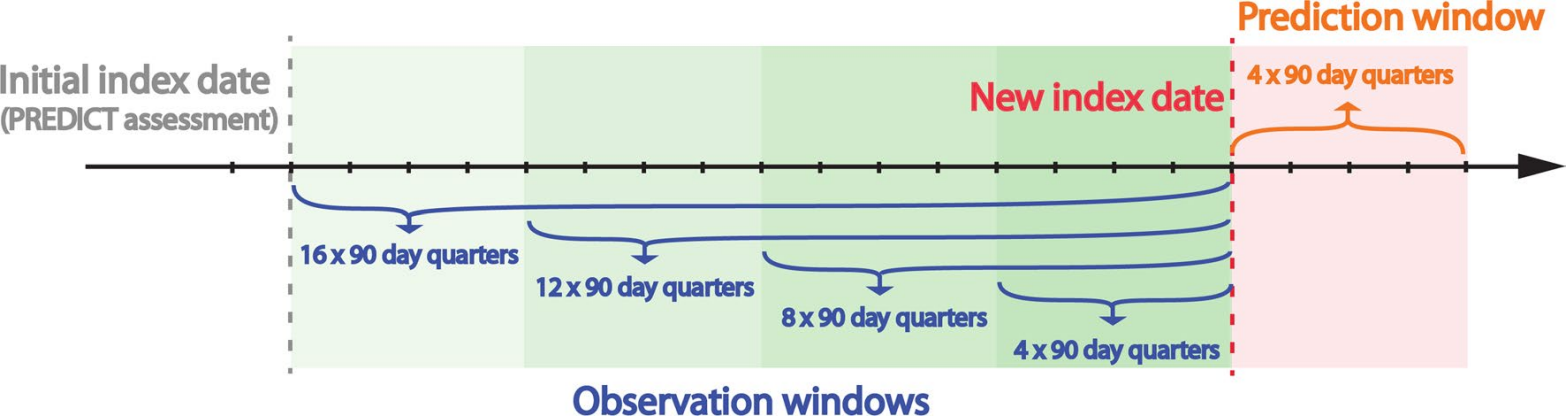
Study design of the Post-PREDICT adherence prediction experiments showing the initial index date, new index date, the observation windows (shaded in green) and the prediction window (shaded in red)

Based on the hypothesis that the integration of patient history through temporal modelling will aid predictive performance, and that LSTM has the capacity to learn to retain relevant and ignore unimportant patient history, a further question is raised: Could lengthening the observation window demonstrate a more distinct advantage of using LSTM? To answer this question, a further set of experiments is conducted. Using data after the PREDICT assessment/index date (20 quarters), a task is formulated to predict the adherence behaviour in the last year (quarter 17 to quarter 20) using 1, 2, 3 and 4 years (4, 8, 12 and 16 quarters) of patient history before and up to quarter 16 (the new index date). See Fig. 7 for an illustration of the study design of this set of experiments. For this task, patients who died before quarter 16 (4897 individuals) are removed from the data. These experiments are from here on referred to as the *Post-PREDICT* adherence prediction experiments. For these experiments, models LSTM, Simple RNN, MLP and RC are compared.

## Results

**Fig. 8:**
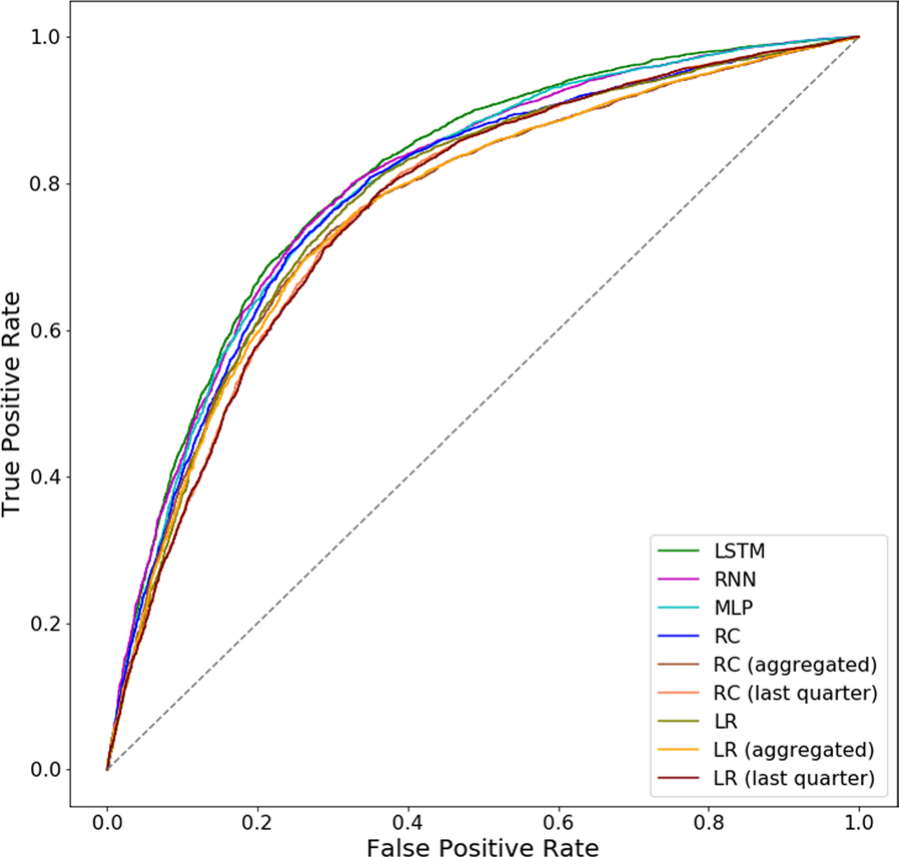
ROC curves of adherence prediction

Table 10 shows the ROC AUC scores of the models on the task of adherence prediction. Figure 8 shows the ROC curves of the models. It can be observed LSTM, Simple RNN, MLP and in some regions RC compare favourably to other model comparators. Over the ROC space, the NN based models dominate over the regression based models with the exception that for a small region RC performed similarly well to MLP. Here, LSTM’s predictive superiority is shown, dominating all other models for the most part of the ROC space. The performance strength of NN models are confirmed in Table 10 where there appears to be a performance gap between NN models and the regression based models, suggesting for this task the capacity to model complex nonlinear relationships is advantageous. Here, the best performing regression models are RC and LR using full sets of features across the observation window. Interestingly, in Fig. 8, the aggregated variant of RC and LR dominates over their last quarter variants in the region where the false positive rate (FPR) is < 0.35, however, where the FPR is > 0.35 the last quarter variants dominate over the aggregated variant.

**Table 10:** Model performance on adherence prediction

Model	ROC AUC
LSTM	0.805
Simple RNN	0.798
MLP	0.794
RC	0.784
RC (aggregated)	0.765
RC (last quarter)	0.766
LR	0.782
LR (aggregated)	0.764
LR (last quarter)	0.765

The results of the pairwise comparison of ROC curves using DeLong’s test are shown in Table 11. The model comparisons of the adherence prediction experiments include 36 hypotheses (pairwise comparison of 9 individual models). The significance level of 0.05 is chosen for determining statistical significance. In Table 11, all p-values below the Bonferroni adjusted significance threshold of 0.00139 are in bold, the Bonferroni adjusted threshold is derived from 0.05/36 as there are 36 pairwise comparisons. Alongside the results shown in Table 10, these results supports our stated hypothesis that an integration of patient history through temporal modelling can aid predictive performance. Here, LSTM and Simple RNN are the top performing models with LSTM shown to perform significantly better than all model comparators. Simple RNN ranking second performed significantly better than all other models except for MLP. The results of the sensitivity analysis are shown in Table 12. The pairwise comparison of ROC AUC scores using Kolmogorov-Smirnov test shows that the models are robust against randomly resampled test sets.

**Table 11:** *p*values of pairwise comparison of models performance on adherence prediction using DeLong’s test

	Simple RNN	MLP	RC	RC (aggr)	RC (last)	LR	LR (aggr)	LR (last)
LSTM	** 3.479e−6**	**5.025e−12**	< **2.2e−16**	< **2.2e−16**	< **2.2e−16**	< **2.2e−16**	< **2.2e−16**	< **2.2e−16**
Simple RNN		0.01353	**7.639e−14**	< **2.2e−16**	< **2.2e−16**	**7.966e−16**	< **2.2e−16**	< **2.2e−16**
MLP			**8.937e−7**	< **2.2e−16**	< **2.2e−16**	**1.538e−8**	< **2.2e−16**	< **2.2e−16**
RC				< **2.2e−16**	< **2.2e−16**	2.741e−3	< **2.2e−16**	< **2.2e−16**
RC (aggr)					0.829	**6.967e−14**	0.02915	0.9102
RC (last)						**3.405e−15**	0.6371	0.001774
LR							**1.666e−15**	< **2.2e−16**
LR (aggr)								0.8881

Using significance level of 0.05, values under the Bonferroni adjusted significance level of 0.00139 are in bold

**Table 12:** *p* values of pairwise comparison of models performance on 1000 (repeats) randomly resampled with replacement test sets (10,096 samples) using Kolmogorov–Smirnov test

	Simple RNN	MLP	RC	RC (aggr)	RC (last)	LR	LR (aggr)	LR (last)
LSTM	** 3.581e−149**	**1.366e−298**	**0.0**	**0.0**	**0.0**	**0.0**	**0.0**	**0.0**
Simple RNN		**5.819e−62**	**0.0**	**0.0**	**0.0**	**0.0**	**0.0**	**0.0**
MLP			**2.441e−255**	**0.0**	**0.0**	**0.0**	**0.0**	**0.0**
RC				**0.0**	**0.0**	**3.683e−19**	**0.0**	**0.0**
RC (aggr)					0.01960	**0.0**	2.029e−3	0.08690
RC (last)						**0.0**	**2.462e−7**	**7.336e−5**
LR							**0.0**	**0.0**
LR (aggr)								0.1641

Using significance level of 0.05, values under the Bonferroni adjusted significance level of 0.00139 are in bold

**Fig. 9:**
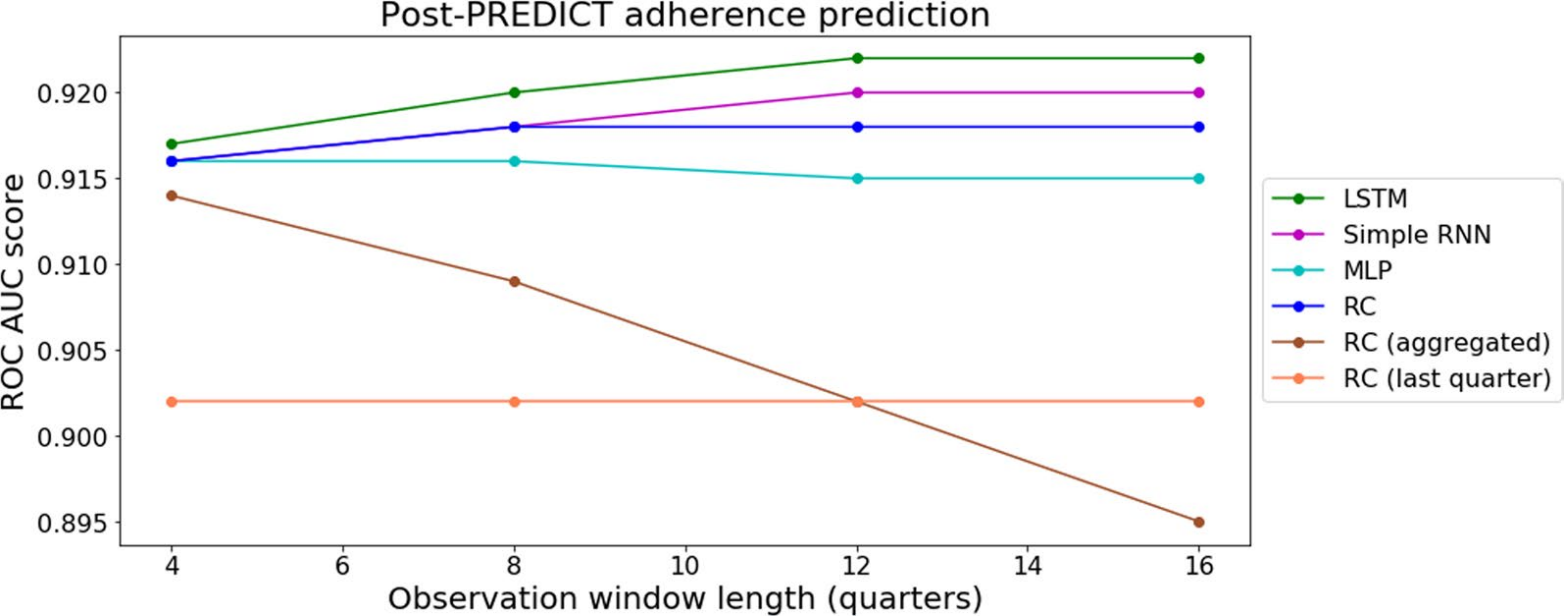
Model performance on the Post-PREDICT adherence prediction with varying observation window lengths

The results of the experiments using data after the PREDICT assessment date with varying lengths of observation window is shown in Fig. 9. The corresponding results of DeLong’s tests are shown in Table 13. Here, it can be observed that as the observation window grew in length, the performance of temporal models LSTM and Simple RNN continued to improve up to 12 quarters. The non-temporal comparator MLP and RC were unable to leverage additional context beyond 8 quarters to improve its prediction. Interestingly, RC (aggregated)’s performance deteriorated with longer observation window. These results confirm that a more distinct advantage of using LSTM can be demonstrated by lengthening the observation window.

## Discussion

The results of the adherence prediction experiments show that for predicting adherence to lipid-lowering medication over a 5-year period based on a 2-year (8 quarters) observation window it is beneficial to use the full sets of features across the observation window. With RC and LR, both models perform significantly better than their aggregated and last quarter counterparts despite the strong presence of multicollinearity. There is also evidence of nonlinear relationships among the predictor variables as shown by the superior performance of MLP in contrast to the linear models. Additionally, the observed ROC AUC of Simple RNN is higher than that of MLP, but the result is not statistically significant with the adjusted significance level (it is worthwhile to mention that the Bonferroni adjustment is conservative and can lead to a high rate of false negatives [83]). Overall, LSTM is the superior model, outperforming all comparators. These results demonstrate that for the problem of adherence prediction, explicitly modelling history sequentially and learning to “remember” and “forget” influential and unimportant events in the past is valuable for predictive performance. Our findings of the strengths of deep learning models when compared with regression based models are consistent with observations in other recent studies in the biomedical domain, including for the task of adherence prediction among hypertensive patients [84], and specifically the advantage of LSTM for the tasks of in-hospital mortality, decompensation, length of stay and phenotyping prediction for ICU patients using clinical time−series data [85].

In our experiments using data after the PREDICT assessment with varying lengths of observation window, it is observed that only certain models’ performance improved with longer observation windows, namely LSTM, Simple RNN and RC (See Fig. 9). Additionally, it can be observed that the method of aggregating data from the past, with equal weight, is detrimental to the prediction of regression based models. From Table 13, results of DeLong’s tests show LSTM and Simple RNN are able to leverage additional contexts of patient history to produce predictions that are statistically significantly better than the performance of MLP (LSTM from 8 quarters and Simple RNN from 12 quarters). For this specific task, 12 quarters of observation window seems to be the most effective context for LSTM, allowing it to perform significantly better than RC. Our findings aligns with other studies that showed the capacity of explicitly sequential models to improve with longer context of observation [86, 87]. However, in contrast to [87] MLP did not benefit from longer observation windows in our experiments, and aggregating data across the temporal dimension negatively impacted the performance of regression based models as shown in RC (aggregated).

**Table 13:** Post-PREDICT adherence prediction

Qtrs		Simple RNN	MLP	RC	RC (aggr)	RC (last)
4	LSTM	0.6711	0.4094	0.648	1.807e−2	< **2.2e−16**
	Simple RNN		0.6883	0.9242	4.674e−2	**2.84e−15**
	MLP			0.7797	0.1005	**1.475e−14**
	RC				2.368e−2	< **2.2e−16**
	RC (aggr)					**2.734e−10**
8	LSTM	9.606e−3	**1.169e−4**	2.48e−2	**5.847e−13**	< **2.2e−16**
	Simple RNN		5.614e−2	0.7944	**2.469e−9**	< **2.2e−16**
	MLP			7.817e−2	**7.099e−5**	**2.448e−11**
	RC				**7.365e−10**	< **2.2e−16**
	RC (aggr)					**1.4444e−3**
12	LSTM	7.864e−2	**2.818e−8**	**2.351e−3**	< **2.2e−16**	< **2.2e−16**
	Simple RNN		**2.207e−4**	0.124	< **2.2e−16**	< **2.2e−16**
	MLP			8.743e−3	**2.936e−11**	**1.16e−9**
	RC				< **2.2e−16**	< **2.2e−16**
	RC (aggr)					0.8463
16	LSTM	6.124e−2	**1.799e−6**	5.112e−3	< **2.2e−16**	< **2.2e−16**
	Simple RNN		**6.84e−4**	0.1974	< **2.2e−16**	< **2.2e−16**
	MLP			9.566e−3	< **2.2e−16**	**2.737e−9**
	RC				< **2.2e−16**	< **2.2e−16**
	RC (aggr)					1.063e−2

Qtrs are the number of quarters in the obervation window. The resulting p-values of pairwise comparison using DeLong’s test. Using significance level of 0.05, values under the Bonferroni adjusted significance level of 3.333e$-$3 are in bold

The results demonstrate the advantage of lengthening the observation window for LSTM and Simple RNN models that take explicitly sequential data as input for the task of adherence prediction. Interestingly, the ability to model nonlinear relationships does not give MLP an advantage over RC. This could be due to the length of the prediction window. To predict adherence behaviour over 1 year is a much easier task than to predict adherence behaviour over 5 years. The results suggest there are complex nonlinear relationships in adherence prediction that become important when the prediction window is longer.

There are a number of limitations of the current study. It is not uncommon in the course of a chronic condition for patients with the condition to also suffer from one or more comorbidities. Some of these comorbidities can have consequential effects on the risk and outcome of the patient. In the case of CVD, diabetes and renal disease are known independent risk factors that contribute to an increased risk of a CVD event [3, 88]. While covariates that indicate the presence of comorbidities are included in the current study (e.g. eGFR and HbA1c) the effects of the combination of multiple drugs or polytherapy and by extension the complexity of treatment regimen on adherence has not been addressed. Additionally, the effects of medication titration and known side-effects of statins such as myopathy, elevated creatine kinase levels, and diabetes [3] have not been explored in the context of adherence prediction. Further, researchers of CVD therapy have pointed to the knowledge gap that exists between the evidence from randomised clinical trails, typically only lasting a few years, and the effect of long-term medication treatment (where it is common for therapy to continue for decades) in secondary prevention [89]. The study design used in this thesis was unable to capture the long-term (defined in the scale of decades) effect of disease progression and treatment trajectory. While preserving a useful number of cases, the data construction used in this thesis was only able to achieve a 7 year window to divide between observation and prediction. In the future, however, this will change as routinely collected electronic health records lengthen year on year. LSTM, like other NN models, is a class of black box models where the influence of and interactions between predictor variables cannot be readily explained. Considerable research has been carried out investigating methods to interpret and explain neural models [90, 91], and some specifically for RNNs such as through the use of an attention mechanism [92] or deriving feature attribution from Learned Binary Masks and KernelSHAP [93]. These methods are clearly worthy directions of future work as they hold the potential for aiding risk communication. Finally, the study was limited to patients in the PREDICT cohort; while PREDICT is widely-used in New Zealand general practice, the cohort is not identical to all users of lipid-lowering therapy in the general population. Without having external validation conducted, the generalisability of the findings are limited.

The current study confirms the hypothesis that integrating patient history through temporal modelling is beneficial to the prediction of medication adherence. This has implications for the monitoring of long-term statin therapy as well as other similar diseases where the management and treatment of the disease is long-term. In this study we have refrained from exploring in depth how the model would be operationalized in clinical decision support (e.g. in terms of specific operating thresholds or forms of alerts). However, the observed ROC AUC of 0.805 on 5-year adherence indicates that 80% of pairings of adherent and non-adherent patients are correctly ordered in terms of predicted risk [94]. Practical medication adherence promotion strategies include reminder packaging, reviewing a patient’s total set of medications and simply practicing more effective communication [95]. The performance of the LSTM model presented herein would provide a reasonably-accurate adherence risk stratification to help prioritise such efforts.

## Conclusions

The current study provides evidence that routinely collected health data can be leveraged for the task of adherence prediction. Our findings show integrating patient history using temporal deep learning models is beneficial to predictive performance. LSTM’s performance compares favourably against other model comparators and is a promising model for identifying individuals within a population who might be at risk of medication non-adherence.

## Data Availability

The data that support the findings of this study are not available from the authors due to the terms on which we accessed them from the VIEW research programme. Researchers interested in using the VIEW datasets in collaboration with the VIEW programme should contact Professor Rod Jackson at rt.jackson@auckland.ac.nz. The VIEW research programme data access procedures incorporate the“Five Safes”principles, an internationally recognised risk assessment framework encompassing safe projects, safe people, safe settings, safe data and safe output [96]. To ensure safe projects are conducted using the VIEW data, a template will be provided to allow completion of a data access proposal (DAP) that outlines the proposed research and the specific data required. The DAP will then be considered by the VIEW Leadership team, which is comprised of senior academics, including Professor Rod Jackson and Associate Professor Matire Harwood who are co-directors of the research programme. The research credentials of the applicant will also be reviewed. If approved, researchers are required to adhere to the VIEW team Code of Practice and to sign a Data Release Agreement form that outlines the conditions for data storage and use that must be adhered to (i.e.“safe people”). In terms of safe settings, source data and research-ready VIEW data are not publicly accessible. Researchers will only be able to access data through a VIEW virtual machine that is separate from the wider University network, is disconnected from the internet and is not accessible by USB. Approved researchers are expected to be physically based at the Section of Epidemiology and Biostatistics at the University of Auckland for a few days at the outset of data access. Subsequently, remote access to data can be arranged. Access to source data is highly restricted and requires authorisation from the VIEW team data manager. With consideration of the“safe data”principle, all data made available to researchers are anonymised; the VIEW team does not have access to identifiable health data and therefore the potential for re-identification of research-ready data is minimised. Finally, including a VIEW team member as a co-investigator for all approved research projects involving VIEW data contributes to safe outputs, as all results are reviewed by at least one VIEW team member for any identifying results such as small counts.

## Abbreviations

ACE: Angiotensin-converting-enzyme
ACS: Acute Coronary Syndrome
AUC: Area under the curve
BMI: Body mass index
CVD: Cardiovascular disease
EHR: Electronic health record
eGFR: Estimated glomerular filtration rate
HbA1c: Glycated haemoglobin
HDL: High-density lipoprotein
HMGCoA: 3-Hydroxy-3-methylglutaryl coenzyme A
ICD-10: International Statistical Classification of Diseases and Related Health Problems, 10th Revision
ICD-10-AM: International Statistical Classification of Diseases and Related Health Problems, 10th Revision, Australian Modification
ICU: Intensive care unit
ISPOR: International Society for Pharmacoeconomics and Outcomes Research
LDL: Low-density lipoprotein
LR: Logistic regression
LSTM: Long short-term memory
MELAA: Middle Eastern, Latin American and African
MLP: Multilayer perceptron
MPR: Medication possession ratio
NHI: National Health Index
NMDS: National Minimum Dataset
NN: Neural network
NZDEP: New Zealand Index of Deprivation
PDC: Proportion of days covered
RC: Ridge classifier
ROC: Receiver operating characteristic
RNN: Recurrent neural network
TC/HDL: Total cholesterol/high-density lipoprotein ratio
TCL: Total cholesterol
TRI: Triglyceride
VIEW: Vascular Informatics using Epidemiology and the Web
